# Longitudinal data on advanced cutaneous squamous cell carcinoma from the Dutch Keratinocyte Cancer Collaborative (DKCC): a nationwide real-world database study

**DOI:** 10.1016/j.lanepe.2025.101501

**Published:** 2025-10-18

**Authors:** Loes M. Hollestein, Celeste J. Eggermont, Marieke W.J. Louwman, Kay Schreuder, Meggie C.M. Drissen, Olivia F.M. Steijlen, Antien L. Mooyaart, Annette H. Bruggink, Quirinus J.M. Voorham, Dirk J. Grunhagen, Mathilde Jalving, Tamar E.C. Nijsten, Marlies Wakkee

**Affiliations:** a Department of Dermatology, Erasmus MC Cancer Institute, University Medical Center, Rotterdam, the Netherlands; b Department of Research and Development, Netherlands Comprehensive Cancer Organization (IKNL), Utrecht, the Netherlands; c Department of Pathology, Erasmus MC Cancer Institute, University Medical Center, Rotterdam, the Netherlands; d Palga Foundation, Houten, the Netherlands; e Department of Surgical Oncology, Erasmus MC Cancer Institute, University Medical Center, Rotterdam, the Netherlands; f Department of Medical Oncology, University Medical Center Groningen, Groningen, the Netherlands

**Keywords:** Nationwide cancer registry, Cutaneous squamous cell carcinoma, Epidemiology, Metastasis, Recurrence, Locally advanced cutaneous squamous cell carcinoma

## Abstract

**Background:**

While Cutaneous Squamous Cell Carcinoma (CSCC) is one of the most common cancers, epidemiological data is scarce. Therefore, the aim of this study was to collect high quality epidemiological data, which is needed to identify differences in outcomes and improve patient care. To achieve this aim, we designed the Dutch Keratinocyte Cancer Collaborative (DKCC), which is a nationwide database, including longitudinal data on locally advanced, recurrent, and metastatic CSCC (advanced CSCC).

**Methods:**

Advanced CSCCs were identified from the Dutch nationwide Pathology Databank (Palga) using a validated algorithm. Manual registration of a selection of 500 patients with advanced CSCC per year (all metastatic, all recurrences and a random selection of locally advanced CSCCs) was performed by the Netherlands Cancer Registry to obtain data on tumour characteristics, diagnostics, disease progression and treatment. Data was linked to the Netherland Organ Transplant Registry to obtain information about immunosuppressed patients and to the municipal records for vital status.

**Findings:**

We estimated that 8.0% (1846/23,065 CSCC, 95% confidence interval: 7.4–8.6) of all diagnosed CSCC in 2021 were locally advanced CSCC. In 2021–2022, 920 patients with advanced CSCC were registered in the DKCC. A quarter of metastatic patients had recurrent CSCC before developing metastasis (i.e., 13/51 patients with skin metastasis, 63/296 patients with regional lymph node metastasis, 20/72 patients with distant metastasis). The median time to recurrence or metastasis after an American Joint Committee on Cancer (AJCC) T3/T4 primary CSCC was 11 months (Interquartile range [IQR] 6–19) and 9 months (IQR 6–17), respectively. In 6% (4/67, skin metastasis only) to 20% (17/83, distant metastasis) of episodes with metastasis, no treatment was provided.

**Interpretation:**

The DKCC is the first nationwide longitudinal data source on advanced CSCC. Its methodology can serve as an example for designing efficient registries for advanced CSCC in other countries or even for other rare cancer outcomes. The high number of locally advanced CSCC puts a large burden on the health care system, as these patients need more extensive work-up and treatment. Data from the DKCC provides essential information to improve clinical guidelines for optimal CSCC patient care.

**Funding:**

Funded by 10.13039/100013995Sanofi Genzyme/10.13039/100009857Regeneron.

**Research in context**

**Evidence before this study**

Most patients with cutaneous squamous cell carcinoma (CSCC) have an excellent prognosis and are cured by surgical excision. However, a subset of tumours progresses locally, recurs, or metastasizes, leading to significant morbidity and mortality. Before this study nationwide data were lacking about: 1) the number of advanced CSCC (i.e., locally advanced, recurrence and metastatic disease), 2) progression of CSCC, 3) treatment patterns and differences in treatment strategies, 4) disease-specific deaths due to each subtype of advanced CSCC. We conducted a literature review using PubMed, Embase, and Scopus with search terms such as “cutaneous squamous cell carcinoma”, “locally advanced”, “recurrence”, “metastasis”, “incidence”, “mortality”, and “epidemiology” up to April 2025. In 2022, two nationwide, population-based studies from the Netherlands and the UK were published, that focused on metastatic CSCC. Most other studies were limited by a retrospective design, single-center scope and inconsistent definitions of advanced disease. Even current staging systems are based on single-center studies.

**Added value of this study**

This study is the first to provide nationwide, real-world longitudinal data on advanced CSCC. By using a validated algorithm in the Dutch nationwide Pathology Databank (Palga) and detailed manual registration through the Netherlands Cancer Registry, we present a reliable estimates of number of patients or tumours of each type of advanced CSCC, disease progression patterns and details of diagnostics, treatment and follow-up of advanced CSCC. The prospective registration of 500 advanced CSCC patients per year since 2021 within the Dutch Keratinocyte Cancer Collaborative (DKCC) offers crucial insights into the timeline of recurrence and metastasis, as well as treatment gaps. These data can be used to contribute to improved evidence-based clinical guidelines for CSCC.

**Implications of all the available evidence**

CSCC places a considerable burden on healthcare systems worldwide, as it is one of the most common cancers with approximately 2 million newly diagnosed patients each year. One third of all patients also develop multiple CSCC. We observed, that locally advanced CSCC accounts for 8% of all first and subsequent CSCC diagnoses each year. Many tumours that recurred or metastasized were initially classified as low-risk, underscoring limitations in current staging methods. Most metastasis occurred during follow-up, but routine lymph node ultrasound for all patients during follow-up is not feasible. These results underline the urgent need for improved prognostic models and evidence-based guidelines for diagnostics, treatment and follow-up to manage the burden of CSCC on the health care systems. Nationwide data from the DKCC will support the development of prognostic tools and help refine evidence-based clinical surveillance strategies and treatment guidelines.

## Introduction

Most patients with cutaneous squamous cell carcinoma (CSCC) have an excellent prognosis and are cured by surgical excision.^1^ However, a subset of tumours progresses locally, recurs or metastasizes, causing significant morbidity and sometimes mortality.^1^, ^2^, ^3^, ^4^ Half of all patients who die due to CSCC did not have histologically confirmed metastasis.^5^ Although some of those patients had non-histologically confirmed metastasis, most likely died due to locally advanced CSCC, because many patients who died due to CSCC had a pathology report of a high-risk locally invasive CSCC(5). The morbidity due to advanced CSCC is high, because patients need extensive surgery to achieve curation, which is often very mutilating (e.g., complete removal of the nose or ears). While population-based data on metastatic CSCC is available, locally advanced and recurrent tumours remain vastly understudied due to a lack of comprehensive population-based data.^3^^,^^5^ Reliable data on the number and characteristics of patients with advanced CSCC is essential for guiding health care policymakers and optimizing patient care.

However, many national cancer registries either exclude CSCC from registration or limit reporting to the first CSCC per patient or per year.^6^, ^7^, ^8^ In contrast, the Netherlands Cancer Registry (NCR) includes all first and subsequent CSCC. Until now, follow-up data on CSCC was not collected, as manual review of all patient records for disease progression is not feasible. Therefore, we developed a rule-based algorithm to efficiently identify advanced CSCC patients at a population-based level.^9^ This approach enabled to collect follow-up information on advanced CSCC on a nationwide level using the linkage between the Dutch Nationwide Pathology Databank (Palga) and the NCR.^10^ As a result, we established the Dutch Keratinocyte Cancer Collaborative (DKCC), a prospective, nationwide registry with longitudinal follow-up for advanced CSCC.

In this study, we aim to^1^ outline the methodology behind the formation of the DKCC,^2^ estimate the annual number of patients with advanced CSCC at a population level, and^3^ provide a comprehensive characterization of patients with advanced CSCC, including tumour characteristics, diagnostics, disease progression and treatments of patients who were included in the DKCC in 2021 and 2022. Inclusion of patients and collection of follow-up data will continue hereafter, which facilitates future survival and comparative analyses.

## Methods

### Setting

Since 1991, Palga retrieves all histopathologically confirmed cases of CSCCs from all Dutch pathology laboratories.^11^ Via the Palga infrastructure, the NCR is notified about all newly diagnosed and subsequent CSCCs, which are registered automatically.^10^

### Identification of advanced CSCC

**Fig. 1:**
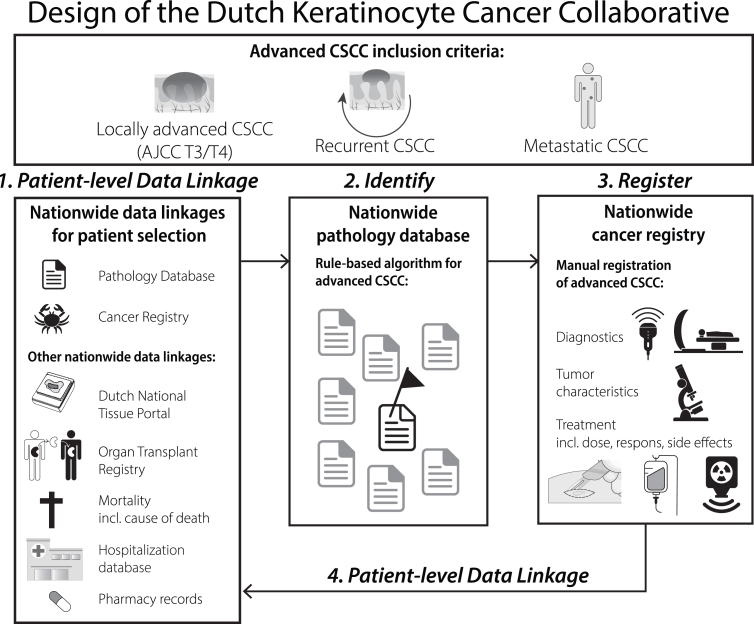
Visual abstract.

Advanced CSCC was defined as locally advanced primary CSCC (either T3/T4 according to the American Joint Committee on Cancer [AJCC 8] or T2b/T3 according to Brigham and Women Hospital [BWH] T-stage classification), recurrent CSCC, or metastatic CSCC (skin, nodal, or distant metastasis) (Fig. 1).^12^^,^^13^ Patients with advanced CSCC were identified from Palga using a validated rule-based algorithm of hierarchical pathology codes and the free-text pathology-report conclusions.^9^ The algorithm enabled efficient identification of advanced CSCC cases from a large number of pathology reports (i.e., 2350 advanced CSCC reports from an annual pool of >30,000 CSCC-related pathology reports). Each selected pathology report was flagged as: ‘metastatic’, ‘recurrence’, ‘locally advanced’ or ‘major surgery’ to enable prioritization of registration. The algorithm demonstrated a sensitivity of 92% and positive predictive value (PPV) of 79%.^9^ During the design phase of the DKCC (in 2017) we estimated that 500 patients would be enough to at least include all metastasis, recurrent CSCC and patients with major surgeries. This estimation was based on data from only 3 centers, which we extrapolated to the entire Dutch population, because there were no population-based studies available at that time.^14^^,^^15^ We prioritized all cases of metastatic and recurrent CSCC for registration, and the remaining capacity was supplemented up to 500 with cases of locally advanced CSCCs (AJCC 8 T3/T4 and/or BWH T2b/T3). We included all locally advanced CSCC cases involving major surgery, such as amputation of the nose, ear or orbital exenteration), as described in the pathology report. We used a random selection process for other primary locally advanced CSCC cases.

Manual verification and registration by the NCR registrars (i.e., trained employees who record items in the NCR based on the medical files) was performed for the selected cases. Inclusion criteria were based on morphology- and topography codes according to the third edition of the International Classification of Diseases for Oncology (ICD-O3) (Supplementary Table S1).^16^

### Data collection

A multidisciplinary advisory board was composed of different types of treating medical specialists including a dermatologist, pathologist, medical oncologist, surgical oncologist, head and neck surgeon and radiotherapist, to guide the selection of the recorded items and to assess the feasibility of the pilot registration (described below).

The DKCC registration manual consisted of a core item set to be recorded (Fig. 1, Supplementary Table S2). These core items were incorporated into the NCR registration system and included patient demographics (e.g., age, sex, performance and vital status), detailed information on primary tumour (e.g., date of diagnosis, imaging, treatment), as well as data on local recurrence (e.g., diagnosis and treatment dates), metastasis (including both histologically and non-histologically confirmed metastasis, type, date, treatment) and systemic therapy outcomes.

### Pilot registration and quality control

Prior to the start of the final registration, we conducted a pilot registration including 500 patients with advanced CSCC diagnosed in 2018 and 2019 (without annual follow-up; data not included). This pilot registration aimed to assess the feasibility of the items to be recorded and to refine the registration manual as needed.

The pilot registration revealed that some NCR registrars had limited experience with CSCC registration. To address this, a dedicated DKCC team of registrars was selected for the final registry. This team received specialized training, including educational sessions on CSCC and detailed instructions on the DKCC manual. In cases of uncertainty, the registrars were encouraged to consult an attending physician-researcher (CE) for clarification. Additionally, periodic cross-checks were implemented, where NCR registrars reviewed and verified each other's work to ensure the accuracy and consistency of the data. Details on the training and quality control are described in the Supplementary Methods.

### Final prospective data collection

After finalization of the pilot registration, prospective inclusion of advanced CSCCs was initiated from January 1st, 2021. The primary CSCC could have occurred before this date. For example, if a patient developed metastasis in 2021 as a result of a primary tumour in 2019, both the primary tumour and the metastasis were registered. Registrars reviewed all DKCC patient records annually to register disease progression or new treatments. As patients were included at different dates, the total follow-up time for each patient will vary. Therefore, the date of the last access of the hospital files was registered. This date will be used in future survival analyses as censoring date to account for variable follow-up durations. When a patient moved or was treated in another hospital, follow-up was complete, because all hospitals in the Netherlands were included.

### Patient-level data linkage

To increase the data granularity and research efficiency (reducing the need for manual registration), the DKCC data can be linked to multiple nationwide routinely collected health care databases. For this descriptive study, the data was linked to the Netherlands Organ Transplant Registry to obtain information on solid organ transplantations and to the municipal records to obtain vital status and date of death (until 1 February 2024). For future analyses, data can also be linked to the cause of death registry of Statistics Netherlands for disease-specific survival analyses; to the Dutch National Tissue Portal to obtain formalin-fixed paraffin-embedded (FFPE) tumour material; to the Dutch Hospital Database for hospitalization data and to pharmacy records.

### Statistical analyses

To estimate the nationwide number of patients of advanced CSCC, we used different methods per type of advanced CSCC. For metastatic and recurrent CSCC we based our estimation on the number of patients included in the DKCC in 2021, as we included all identified patients on a nationwide level. We estimated the annual number of patients instead of incidence rates, as we included both incident and prevalent CSCC patients with metastasis and recurrences. The algorithm's sensitivity and PPV was used to estimate the annual number of patients in the total population (Supplementary Methods and Supplementary Table S3).^9^ For locally advanced CSCC we included a random sample in the DKCC. Therefore, we decided it would be more accurate to use nationwide pathology records for these calculations instead of this random sample. We estimated the nationwide number of newly diagnosed locally advanced primary CSCC. We analysed all pathology reports identified by the rule-based algorithm for advanced CSCC in 2021 among all synoptic reports and used the algorithm's sensitivity to estimate the nationwide occurrence of advanced CSCC (Supplementary Methods and Supplementary Table S3).^9^

We describe the characteristics of all patients included in DKCC 2021–2022, stratified by type of advanced CSCC. Criteria of locally advanced primary CSCC for both AJCC and BWH T-stage are provided in Supplementary Table S4 Descriptive statistics were performed using SPSS 28.0 (SPSS Inc) and SAS 9.4 (SAS Institute).

### Ethics approval

This study received approval from the scientific committees of the NCR (K22.112), Palga (lzv2021-145), Erasmus Medical Center (MEC-2020-0054), and Dutch Clinical Research Foundation (W20.048/NWMO20.02.007) and a waiver of informed consent was granted.

### Role of the funding source

The funders had no role in study design, data collection, data analysis, data interpretation or writing of the manuscript.

## Results

### Estimated nationwide number of advanced CSCC

In 2021, 14,700 patients were newly diagnosed with CSCC and a total of 23,065 CSCCs (including both primary and subsequent CSCC) were registered in the Netherlands, which has a population of 17.5 million people (Supplementary Table S5). Using data from DKCC, we estimated that in the Netherlands 257 (95% confidence interval [CI]: 214–321) patients were diagnosed with a metastasis, 521 (95% CI: 311–1500) with recurrent CSCC (any AJCC T-stage) and 1754 patients (95% CI: 1621–1885) with locally advanced CSCC (AJCC T3/T4). These AJCC T3/T4 locally advanced CSCCs represent 8.0% (N = 1,846, 95% CI: 7.4%–8.6%) of all 23,065 histopathologically-confirmed CSCCs in 2021. As one of the risk factors of BWH-staging (i.e., invasion beyond subcutaneous fat) was not routinely registered in the Netherlands, we assumed, that an invasion depth greater than 6 mm was invasion beyond the subcutaneous fat to estimate the proportion of CSCC cases annually diagnosed as BWH T2b/T3. We estimated that the proportion of BWH T2B/T3 locally advanced CSCC was 8.8% (N = 2,036, 95% CI: 7.1%–11.5%) of all histopathologically-confirmed CSCC in 2021.

### Longitudinal registration of advanced CSCC

**Fig. 2:**
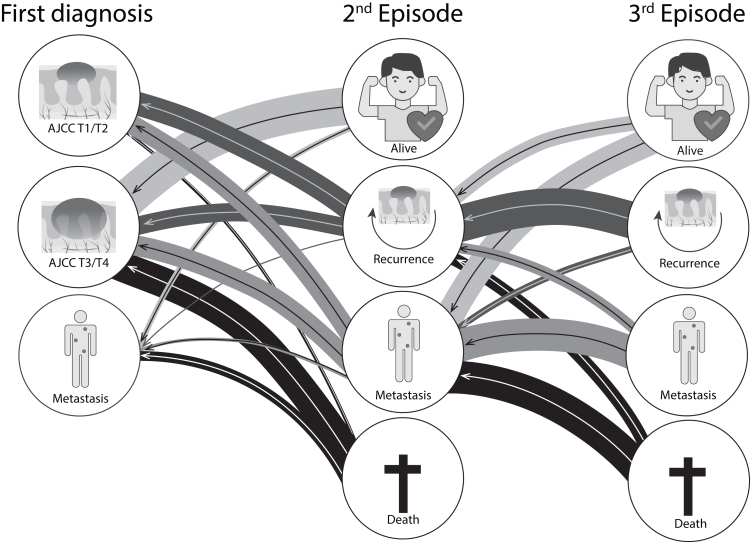
The Patient Journey of advanced CSCC patients—preceding CSCC stages. This figure represents the patient journey and shows which CSCC preceded each CSCC stage. The patient journey in this figure should be read from right (last episode) to left (what came before?), because in the DKCC was registered what type of CSCC occurred before an advanced CSCC, rather than registering the progression of all CSCC. The thickness of the lines represent the proportion of patients that move from one stage to another. The state on the right side of each line counts as 100%. The percentage associated with each line is described in Supplementary Table S6.

In 2021 and 2022, DKCC included 929 patients. After excluding 9 patients with an unknown primary CSCC, 920 patients were available for analyses. The DKCC provides detailed insights into the patient journey and which CSCC stage preceded advanced CSCC (Fig. 2, Supplementary Table S6). Many patients with recurrences or metastasis were initially diagnosed with low-risk CSCC (AJCC T1/T2). For example, of patients who developed metastasis as a second episode, equal proportions were initially diagnosed with low risk T1/T2 CSCC (46%) or high risk T3/T4 CSCC (46%).

We observed multiple metastatic patterns (Supplementary Table S7). The majority (277/351, 77%) of metastatic patients developed metastasis during follow-up.

### Locally advanced and recurrent CSCC

A total of 559 primary locally advanced CSCC (AJCC8 T3/T4) and 263 recurrent CSCCs were included (Table 1). These 263 recurrences included CSCC that recurred multiple times. We identified 198 primary CSCCs from which the (re-)recurrences originated. Although risk factors required to calculate BWH staging are not routinely recorded in the Netherlands, 278 of 559 T3/T4 tumours could also be classified as BWH stage T2b/T3. The median age was 80 or higher for each group, with the majority being male (70–73%). Most primary locally advanced CSCCs were treated with surgery alone (512/559, 92%), while this was 76% (201/263) for recurrent CSCC. Compared to primary locally advanced CSCC, recurrent CSCCs were more often treated with curative intent radiotherapy, (6% versus 1%, 17/263 versus 7/559), surgery with adjuvant radiotherapy (9% versus 6%, 23/263 versus 35/559) and systemic treatment (4% versus 1%, 10/263 versus 3/559). More than a quarter of all primary locally advanced (131/507) and recurrent CSCC (56/197) were incompletely excised (i.e., after the last (re-)excision), whereafter 25% (18/71)–41% (23/56)) received an additional treatment, such as radiotherapy or margin-controlled surgery (Table 1, Supplementary Table S8). An incompletely excised primary tumour of a recurrence only received additional treatment in 20% (10/51) of all cases. The median time to recurrence or metastasis after a AJCC T3/T4 CSCC was 11 months (interquartile range [IQR] 6–19) and 9 months (IQR 6–17), respectively.

**Table 1:** Primary locally advanced CSCC and recurrent CSCC.

Characteristics	Primary locally advanced CSCC		Recurrent SCC	
	AJCC8 T3/T4	BWH T2b/T3	Primary CSCC of the recurrence	Recurrence
	n = 559	n = 278	n = 198^a^	n = 263
**Patient characteristics**				
**Sex**				
Male	392 (70%)	203 (73%)	128 (65%)	167 (63%)
Female	167 (30%)	75 (27%)	70 (35%)	96 (37%)
**Age, median (IQR), y**	81 (74–86)	82 (75–86)	80 (73–85)	82 (75–86)
**Immunosuppression**				
Hematological malignancy	57 (10%)	38 (14%)	31 (16%)	42 (16%)
Organ transplant recipient	47 (8%)	24 (9%)	20 (10%)	27 (10%)
**Number of previous CSCC**				
None	274 (49%)	139 (50%)	104 (53%)	NA
1	112 (20%)	46 (17%)	46 (23%)	NA
2–4	138 (25%)	71 (26%)	35 (18%)	NA
≥5	35 (6%)	18 (6%)	13 (7%)	NA
**Tumour characteristics**				
**Localization**				
Head/neck	381 (68%)	197 (71%)	145 (73%)	195 (74%)
Lip, ear	68 (12%)	30 (11%)	23 (12%)	30 (11%)
Trunk	37 (7%)	18 (6%)	2 (1%)	2 (1%)
Arm/hand	44 (8%)	19 (7%)	10 (5%)	11 (4%)
Leg/foot	23 (4%)	11 (4%)	15 (8%)	20 (8%)
Other localizations	6 (1%)	3 (1%)	3 (2%)	5 (2%)
**Size**^b^				
<2 cm	253 (45%)	61 (22%)	122 (62%)	139 (53%)
≥2 cm and <4 cm	227 (41%)	181 (65%)	45 (23%)	60 (23%)
≥4 cm	65 (12%)	34 (12%)	11 (6%)	19 (7%)
*Not reported*	14 (3%)	2 (1%)	20 (10%)	45 (17%)
**Differentiation**				
Well	88 (16%)	23 (8%)	39 (20%)	46 (17%)
Moderate	290 (52%)	108 (39%)	94 (47%)	122 (46%)
Poor	131 (23%)	137 (49%)	44 (22%)	74 (28%)
*Not reported*	50 (9%)	10 (4%)	21 (11%)	21 (8%)
**Depth of invasion**				
≤6 mm	129 (23%)	86 (31%)	117 (59%)	105 (40%)
>6 mm	338 (60%)	128 (46%)	39 (20%)	65 (25%)
*Not reported*	92 (16%)	64 (23%)	42 (21%)	93 (35%)
**Invasion beyond fat**				
No	160 (29%)	57 (21%)	98 (49%)	99 (38%)
Yes	215 (38%)	166 (60%)	30 (15%)	75 (29%)
*Not reported*	184 (33%)	55 (20%)	70 (35%)	89 (34%)
**Perineural invasion**				
No	232 (42%)	97 (35%)	105 (53%)	152 (58%)
Yes				
Nerves <0.1 mm	7 (1%)	3 (1%)	2 (1%)	3 (1%)
Nerves ≥0.1 mm	19 (3%)	12 (4%)	0 (0%)	9 (3%)
Deep nerves	23 (4%)	19 (7%)	4 (2%)	5 (2%)
Depth unknown	132 (24%)	86 (31%)	24 (12%)	34 (13%)
*Not reported*	146 (26%)	61 (22%)	63 (32%)	60 (23%)
**Lymphovascular invasion**				
No	480 (86%)	236 (85%)	171 (86%)	210 (80%)
Yes	49 (9%)	31 (11%)	8 (4%)	9 (3%)
*Not reported*	30 (5%)	11 (4%)	19 (10%)	44 (17%)
**T-stage AJCC 8**				
T1	NA	0 (0%)	83 (42%)	77 (29%)
T2	NA	24 (9%)	18 (9%)	20 (8%)
T3	553 (99%)	248 (89%)	79 (40%)	126 (48%)
T4	4 (1%)	5 (2%)	1 (1%)	3 (1%)
*Cannot be computed*	1 (0%)	1 (0%)	17 (9%)	37 (14%)
**T-stage BWH**				
T1	18 (3%)	NA	27 (14%)	33 (13%)
T2a	73 (13%)	NA	24 (12%)	37 (14%)
T2b	141 (25%)	150 (54%)	24 (12%)	44 (17%)
T3	16 (3%)	16 (6%)	1 (1%)	12 (5%)
*Cannot be computed*^c^	311 (56%)	112 (40%)	122 (62%)	137 (52%)
**Treatment**				
**Surgery monotherapy**	512 (92%)	254 (91%)	182 (92%)	201 (76%)
Of which staged excision^d^	50 (9%)	23 (8%)	13 (7%)	23 (9%)
Of which major surgery^e^	40 (7%)	24 (9%)	2 (1%)	10 (4%)
**Surgery + adjuvant radiotherapy**	35 (6%)	19 (7%)	11 (6%)	23 (9%)
Of which staged excision^d^	2 (0%)	2 (1%)	0 (0%)	6 (2%)
Of which major surgery^e^	4 (1%)	4 (1%)	1 (1%)	2 (1%)
**Primary curative intent radiotherapy**	7 (1%)	3 (1%)	4 (2%)	17 (6%)
**Systemic treatment**	3 (1%)	1 (0%)	1 (1%)	10 (4%)
With staged excision^c^ or major surgery^e^	0	0	0	2 (1%)
**No treatment**	2 (0%)	1 (0%)	0	9 (3%)
*Not reported*	0	0	0	3 (1%)
**Margin status**				
Treated with excision, N	507	248	184	197
Negative	336 (66%)	158 (64%)	95 (52%)	127 (64%)
Positive	131 (26%)	71 (29%)	51 (28%)	56 (28%)
Additional treatment	42 (32% of 131)	18 (25% of 71)	10 (20% of 51)	23 (41% of 56)
*Not reported*	40 (8%)	19 (8%)	38 (21%)	14 (7%)
**Time to progression**				
Time to last access date medical file in months (median, IQR)	13 (6–21)	13 (6–21)	25 (16–42)	11 (5–20)
**Local recurrence, N**	74	34	198	53
Time to local recurrence in months (median, IQR)	11 (6–19)	10 (6–24)	14 (7–24)	8 (5–14)
**Metastasis, N**	132	76	38	27
Time to metastasis in months (median, IQR)	9 (6–17)	8 (6–15)	14 (7–24)	9 (6–24)
**Death, any cause, N**	121	61	50	65
Time to death in months (median, IQR)	14 (8–21)	12 (7–20)	22 (12–37)	24 (15–37)

Abbreviations: CSCC, cutaneous squamous cell carcinoma; AJCC 8, American Joint Committee of Cancer, 8th edition; BWH, Brigham and Women Hospital staging system; NA, not applicable.

a As tumours may recur multiple times the number of primary tumours (n = 198) is lower than the number of episodes of recurrences (n = 263).

b This primarily concerns the clinically measured size. If the clinical size was missing the pathological size was used.

c Invasion beyond subcutaneous fat was not routinely reported. We only assigned BWH high-risk features in case it was explicitly mentioned in the pathology report (see Supplementary Table S4 for exact criteria).

d Staged excision includes Mohs micrographic surgery and Breuninger/slow Mohs surgery technique.

e Major surgery includes auriculectomy, rhinectomy, digit amputation, wedge excision or subtotal excision of the lip, orbital exenteration or frasing/resection of the bone.

### Metastatic CSCC

A total of 478 metastatic episodes were analyzed, comprising 67 episodes of skin metastasis, 328 of regional lymph node metastasis, and 83 with distant metastasis (Table 2). Most metastasis were diagnosed during follow-up. A quarter of all metastatic patients had a recurrent CSCC either prior to or concurrently with the metastasis (i.e., 13/51 patients with skin metastasis, 63/296 patients with regional lymph node metastasis, 20/72 patients with distant metastasis). Regional lymph node and distant metastasis were primarily detected because of patient-reported symptoms (238/328, 71% and 59/83, 73%, respectively), whereas skin metastases were more frequently found during routine surveillance (27/67, 40%) (Table 2). In 59% (205/[328 + 20]) of all episodes with regional lymph node metastasis, PET-CT or CT thorax scans were performed to detect distant metastasis [data not shown], though distant metastasis were detected in only 6% (20/[328 + 20]) of these episodes.

**Table 2:** Metastatic CSCC–diagnosis.

Characteristics	Skin metastasis	Regional lymph node metastasis	Distant metastasis
**Nr episodes**	67	328	83
**Nr patients**	51	296	72
**Nr episodes per patient**			
1	41	268	61
2	6	25	11
>2	4	3	0
**Patient characteristics**			
**Sex**			
Male	42 (82%)	223 (75%)	51 (71%)
Female	9 (18%)	73 (25%)	21 (29%)
Age at time of metastasis, median (IQR), y	81 (74–86)	80 (73–86)	81 (72–88)
**Immunosuppression**			
Hematological malignancy	6 (12%)	38 (13%)	12 (17%)
Organ transplant recipient	8 (16%)	25 (8%)	13 (18%)
**Diagnostics**			
**Reason for diagnostics**			
Patient had symptoms	33 (49%)	238 (73%)	59 (71%)
Screening	0 (0%)	2 (1%)	0 (0%)
Surveillance	27 (40%)	65 (20%)	19 (23%)
Co-incidence	1 (1%)	4 (1%)	0 (0%)
Other	1 (1%)	1 (0%)	0 (0%)
Unknown	5 (7%)	18 (5%)	5 (6%)
**Type of diagnostics**			
Ultrasound	32 (48%)	238 (73%)	38 (46%)
CT, neck	9 (13%)	65 (20%)	13 (16%)
CT, thorax	9 (13%)	73 (22%)	27 (33%)
CT, other	11 (16%)	52 (16%)	31 (37%)
MRI	6 (9%)	123 (38%)	20 (24%)
PET/PET-CT	14 (21%)	136 (41%)	37 (45%)
**Characteristics of this episode**			
**Type of episode**			
First CSCC diagnosis	7 (10%)	71 (22%)	11 (13%)
Recurrent CSCC with metastasis	8 (12%)	49 (15%)	14 (17%)
Subsequent episode with metastasis	52 (78%)	208 (63%)	58 (70%)
**Regional Lymph node metastases**			
Yes	NA	328 (100%)	20 (24%)
No	67 (100%)	NA	63 (76%)
**Distant metastases**			
**Nr of metastases per episode**			
1			42 (51%)
2			17 (20%)
3			15 (18%)
>3			9 (11%)
**Organ type**^a^			
Skin			21 (25%)
Lung			30 (36%)
Bone			25 (30%)
Liver			4 (5%)
Brain			1 (1%)
Lymph node, certainly distant			48 (58%)
Lymph node, possibly distant			5 (6%)
Other			11 (13%)
**Previous and subsequent episodes of these patients**			
**Metastasis-free at first date of CSCC diagnosis**	43 (84%)	222 (75%)	57 (79%)
**T-stage AJCC 8 metastasis-free at first date of CSCC diagnosis**			
T1	11 (26%)	81 (36%)	25 (44%)
T2	4 (9%)	29 (13%)	8 (14%)
T3	26 (60%)	102 (46%)	20 (35%)
T4	1 (2%)	1 (0%)	0 (0%)
*Cannot be computed*	1 (2%)	9 (4%)	4 (7%)
**T-stage BWH metastasis-free at first date of CSCC diagnosis**			
T1	4 (9%)	29 (13%)	12 (21%)
T2	5 (12%)	30 (14%)	5 (9%)
T3	10 (23%)	35 (16%)	5 (9%)
T4	2 (5%)	4 (2%)	1 (2%)
*Cannot be computed*	22 (51%)	124 (56%)	34 (60%)
**Recurrent CSCC before or at the same time of metastasis**	13 (25%)	63 (21%)	20 (28%)
**First metastatic episode**			
Skin metastasis	38 (75%)	5 (2%)	3 (4%)
Lymph node metastasis	13 (25%)	288 (97%)	25 (35%)
Distant metastasis	0 (0%)	3 (1%)	44 (61%)
**Time from metastasis-free primary CSCC to:**			
**Skin metastasis, N (%)**	43 (84%)	15 (5%)	5 (7%)
Months (median, IQR)	9 (6–19)	17 (9–19)	17 (15–18)
**Lymph node metastasis, N (%)**	15 (29%)	222 (75%)	23 (32%)
Months (median, IQR)	12 (5–19)	11 (7–21)	10 (5–14)
**Distant metastasis, N (%)**	5 (10%)	23 (8%)	57 (79%)
Months (median, IQR)	19 (11–24)	17 (13–25)	14 (8–25)

Abbreviations: CSCC, cutaneous squamous cell carcinoma; NA, not applicable.

a Adds up to >100%, because patients can have metastases at multiple sites.

Among patients with metastasis at initial CSCC diagnosis, almost half of the primary CSCC were treated with surgery, often combined with radiotherapy, especially in case of lymph node metastasis (32/74, 43%) (Table 3). Skin metastases were most frequently treated with metastasectomy (31/67, 46%), while lymph node metastasis only were most frequently treated with lymph node dissection combined with radiotherapy (141/328, 43%). Systemic therapy, mainly with cemiplimab, was the main treatment for distant metastasis (28/83, 34%) and was occasionally applied for recurrent CSCC (Supplementary Table S9). Response rates and reasons for discontinuing systemic treatment are reported in Supplementary Table S9.

**Table 3:** Metastatic CSCC–treatment.

Characteristics	No (%)	No (%)	No (%)
	Skin metastasis	Regional lymph node metastasis	Distant metastasis
**Treatment primary CSCC, no metastasis at first diagnosis**			
N patients	43	222	57
Surgery monotherapy	37 (86%)	205 (92%)	56 (98%)
Surgery + adjuvant radiotherapy	4 (9%)	13 (6%)	1 (2%)
Primary curative radiotherapy	2 (5%)	3 (1%)	0
No treatment	0	1 (0%)	0
**Treatment primary CSCC with metastasis at first diagnosis (any type of metastasis)**			
N patients	8	74	15
Surgery monotherapy primary tumour	1 (13%)	5 (7%)	3 (20%)
Surgery + radiotherapy	2 (25%)	32 (43%)	5 (33%)
Surgery primary tumour + metastasectomy	1 (13%)	18 (24%)	1 (7%)
Metastasectomy alone	1 (13%)	3 (4%)	0
Primary curative radiotherapy	3 (38%)	8 (11%)	4 (27%)
Systemic treatment	0	5 (7%)	1 (7%)
No treatment	0	3 (4%)	1 (7%)
**Treatment of metastasis**			
N episodes	67	328	83
**No treatment metastasis**			
No treatment	4 (6%)	31 (9%)	17 (20%)
Active surveillance	0	1 (0%)	0
Treatment of only primary tumour	1 (1%)	7 (2%)	4 (5%)
*Unknown treatment*	0	4 (1%)	2 (2%)
**Treatment skin metastasis**			
Metastasectomy	31 (46%)	9 (3%)	0
Radiotherapy	13 (19%)	2 (1%)	3 (4%)
Surgery + radiotherapy	13 (19%)	0	0
**Treatment lymph node metastasis**			
Lymph node dissection		75 (23%)	8 (10%)
Radiotherapy		52 (16%)	13 (16%)
Lymph node dissection + radiotherapy		141 (43%)	7 (8%)
**Elective nodal radiotherapy**	2 (3%)	44 (13%)	1 (1%)
**Surgical and radiological treatment distant metastasis**			
Metastasectomy	NA	NA	4 (5%)
Radiotherapy	NA	NA	7 (8%)
Surgery + radiotherapy	NA	NA	0
**Systemic therapy**			
Systemic therapy, cemiplimab	5 (7%)	12 (4%)	24 (29%)
Systemic therapy, other	0	8 (2%)	4 (5%)
**Aim of radiotherapy**			
All episodes with radiotherapy	26	195	27
Curative	14 (54%)	118 (61%)	5 (19%)
Palliative	4 (15%)	31 (16%)	12 (44%)
*Not described*	8 (31%)	46 (24%)	10 (37%)

Abbreviations: CSCC, cutaneous squamous cell carcinoma; NA, not applicable.

In 6% (4/67, skin metastasis only) to 20% (17/83, distant metastasis) of episodes, no treatment was provided. Reasons included: comorbidities, rapid progression and refusal by patient or family [data not shown]. Palliative radiotherapy was applied in 6% (4/67)–14% (12/83) of all episodes with metastasis.

## Discussion

Here we describe the results of the first nationwide real-world data collection on advanced CSCC. In a population of 17.5 million people with 14,700 newly diagnosed CSCC patients, we calculated that each year 257 patients are diagnosed with metastatic CSCC, 521 patients with recurrent CSCC and 1754 patients with locally advanced CSCC. Locally advanced CSCC accounts for 8% of all first and subsequent CSCC diagnoses each year. Since these patients need more extensive work-up and treatment, this puts a large burden on the health care system.

Although CSCC is one of the most common cancers, epidemiological data on advanced CSCC is scarce.^17^, ^18^, ^19^ In the US a real-world analysis of costs, treatment patterns and outcomes of patients with metastatic CSCC using claims data was performed.^17^ The ADOreg (Arbeitsgemeinschaft Dermatologische Onkologie Registry) in Germany includes health care data of >400 CSCC until now, which are mainly locally advanced (i.e., at least one of the T3/T4 AJCC8 risk factors) or metastatic CSCC and multiple CSCC of those patients.^18^ Data of 39 patients who received immunotherapy has been published.^18^ The recently implemented EURO-NMSC registry-based cohort study aims to include 400 high-risk resected CSCC and 500 non-resectable and metastatic CSCC across Europe.^19^ These studies provide valuable insights in costs and treatment of CSCC, but claims data do not include sufficient details (e.g., histopathological features are not included) and the aforementioned registries are mainly conducted in CSCC expert centers. Therefore these registries do not reflect CSCC care in a nationwide setting. Compared to ADOreg and EURO-NMSC registry, the DKCC includes all identified recurrent and metastatic CSCC, but also does not include all identified locally advanced CSCC. However, in the DKCC the included locally advanced CSCC are a random sample from nationwide pathology records and also includes the care pathway of individual patients who were treated in multiple hospitals (if applicable).

Through the DKCC, we created valuable insights into the progression and care pathways of advanced CSCC from the culprit primary CSCC onwards for all types of advanced CSCC. Results on CSCC progression and future analyses on associations between patient and tumour characteristics and outcomes are likely to be generalizable to CSCC patients from other populations. Care pathways and clinical guidelines differ between different countries. Therefore, the distribution of treatments may differ compared to other countries and the other registries, such as ADOreg or EURO-NMSC.

We identified several issues in CSCC care that should be improved, such as early identification of recurrent tumours and detection of lymph node metastasis during follow-up. Notably, 55% of all recurrent tumours and 46% of those who developed metastasis initially presented as a low risk T1/T2 tumour, with larger proportions of T1 than T2 tumors. Similar patterns were observed in another study based on two nationwide cohorts.^20^ Venables et al. showed that the PPV of all staging systems was suboptimal (ranging from 5 to 13%), indicating that current staging systems are insufficient to identify high-risk patients.^20^

Enhancing risk stratification to identify patients at high risk for recurrence and metastasis could facilitate use of adjuvant treatment or closer monitoring.^21^ In accordance with other studies, we observed that positive margins are an import risk factor for recurrence.^14^^,^^22^ On average, 4% of all CSCC are incompletely excised, but 28% (51/184) of the primary tumours that recurred in our study were incompletely excised, while only a minority (20%) received an additional treatment.^14^^,^^22^^,^^23^ In accordance with prior observations we also noticed that lymph node metastases are mainly detected during follow-up with a median time until detection of 11 months (IQR 7–21), while ultrasound examination of lymph nodes being standard practice only at diagnosis for T3/T4 CSCC in the Netherlands.^24^ However, implementing (additional) ultrasound examinations for all CSCC patients is not feasible within most healthcare systems. Therefore, prognostic models may support identifying the appropriate patients for more intensive monitoring.^25^^,^^26^

To treat inoperable or metastatic CSCC, cemiplimab was approved by the European Medicines Agency in 2019. The Dutch healthcare authorities provided controlled access from 2021 via the DRUG Access Protocol to allow an additional health technology assessment in a prospective real-world setting.^27^ Our study provides the first data on the uptake of cemiplimab in a nationwide setting we showed that cemiplimab was applied in 20% of episodes with distant metastasis and to a few patients with locally advanced or recurrent CSCC. Most likely, this number is higher, because not all locally advanced and recurrent CSCC were included in the DKCC. As 8% of all locally advanced CSCC patients still underwent major surgery, such as amputation of the nose, ear or digit, or orbital exenteration, it is expected that the application of cemiplimab will increasingly expand to neoadjuvant application in case of functional inoperable CSCC due to its demonstrated effectiveness and safety.^28^ Future DKCC data will shed light on the evolving role of cemiplimab in these cases.

Strengths of the DKCC include the longitudinal and nationwide data collection, efficient identification of advanced CSCC with a validated pathology record algorithm, the use of multiple nationwide linkages, a scientific advisory board, trained registrars and quality controls.^9^ The algorithm that we used to identify advanced CSCC patients from the pathology reports has been validated on Dutch pathology reports from the same nationwide pathology database (Palga). Therefore, the algorithm is valid when applied to Dutch pathology reports and has a sensitivity of 92% for all advanced CSCC combined when applied to these data.^9^ While the Dutch algorithm has been translated to international SNOMED-CT codes and English text, external validation studies are needed before this algorithm can be used in other countries. The PPV of 79% was determined on pathology data with a similar prevalence of advanced CSCC.^9^ The percentage of false positives (21%) among all pathology reports that were flagged as ‘advanced CSCC’ by the algorithm did not lead to selection bias, but the PPV was important for an efficient registration process. The reason for this, is that every pathology report that was flagged by the algorithm as ‘advanced CSCC’ was manually checked by NCR registrars. A high PPV reduces the required amount of manpower. The PPV of 79% assured that 8 out of 10 patient files that were manually checked were truly advanced CSCC, that needed to be registered in the DKCC database. This was deemed sufficiently high.

In our previous validation study of the algorithm, we observed that ‘recurrence’ was frequently not mentioned in the pathology report. Therefore, the sensitivity of the algorithm for recurrences separately was only 23%. However, most recurrences were identified by the algorithm as ‘locally advanced CSCC’, because these recurrences frequently have high risk features, such as deep invasion or moderate/poor differentiation. Despite the fact that we captured recurrences in another way, we assume that we missed many recurrences. This can also be seen from the wide 95% CI of the number of patients with recurrences: we included 118 recurrences in 2021, but we estimate that there were most likely between 311 and 1500 patients with a recurrence in this calendar year. The other category of patients that may have been missed by using our pathology algorithm are patients that were diagnosed with metastasis based on imaging only without histopathological confirmation.^9^ In most cases these patients were also identified because they had a primary CSCC with high risk features. The number of patients with metastasis that may have been missed is much lower than the amount of patients with a recurrence that may have been missed, as 70% of all patients with a metastasis, also had a pathology report of a metastasis.^9^

The estimation of the number of locally advanced CSCC on a nationwide level was limited by manual review of only the synoptic reports (∼56% of all reports). We could not use all pathology reports, because without synoptic reporting we could not retrieve all tumour characteristics to determine the tumour stage. Synoptic reports were less frequently used by university hospitals and more frequently used in general hospitals. As a consequence, it is likely, that synoptic reports included a lower proportion of high-risk CSCC compared to all reports. Therefore, our nationwide estimates may be biased towards a lower proportion of locally advanced CSCC compared to the true proportion of locally advanced CSCC in the total population.

Another strengths of this unique data collection is that the DKCC will contribute to filling important knowledge gaps in CSCC literature For example, in this manuscript we describe the number of patients of the different types of advanced CSCC on a nationwide level. Examples of future perspectives include research into factors that contribute to local and systemic progression, prognostic models for disease-specific death, and the impact of the introduction of immunotherapy at population-level.

Missing data on a few high-risk tumour characteristics of primary CSCC could be a limitation for future analyses. Missingness was either due to not using synoptic reporting by the pathologist or the use of AJCC staging instead of BWH staging in the Netherlands. More than half of all pathology reports were synoptic reports, which were reported according to a standard protocol and thus included all important tumour characteristics for AJCC staging. In the pathology reports where synoptic reporting was not used, the pathologist did not report whether or not the high risk features were observed. Most likely the high risk feature was not present if it was not reported by the pathologist. The amount of missing data ranged from 0% to 26% for most tumor characteristics, but was higher for ‘invasion beyond subcutaneous fat’ and ‘BWH T-stage’. The reason for this is that AJCC is used in the Netherlands and one BWH high risk feature is therefore not routinely reported (i.e., invasion beyond subcutaneous fat). The other BWH risk factors are included in synoptic reports. In future analyses of data from DKCC these missing data will be taken into account properly (e.g., by applying multiple imputation methods).

Not all locally advanced primary CSCC could be included in the DKCC, but the random selection assured that all subgroups of locally advanced primary CSCC were included and no selection based on treatment or tumour characteristics was made. We also included all primary tumours of patients that developed metastasis or a recurrence and it is likely that these primary tumours have the highest risk features. Therefore the distribution of the tumour characteristics among our total sample of locally advanced CSCC most likely does not reflect the distribution of these characteristics among all locally advanced CSCC in the population. This means that this distribution cannot be regarded as a population-based distribution of tumour characteristics, but as all subgroups of locally advanced primary CSCC were included by using a random selection, valid analyses can be performed in the future to investigate the association between these tumour characteristics and risk of progression.

To our knowledge, the DKCC is the first real-world nationwide longitudinal data source on advanced CSCC. To decrease the burden on the healthcare system due to the high and rising incidence of CSCC, accurate prognostic models for advanced CSCC are needed to identify which patients would benefit most from specific diagnostics or treatments at different stages. Data from the DKCC will contribute to this aim and support establishing evidence-based guidelines for diagnostics, treatment, and follow-up of advanced CSCC.

## Contributors

LH, ML, AB, QV, TN and MW conceived the study design and methodology. LH, TN and MW acquired funding. AM, DG, MJ, and MW served on the DKCC advisory board. LH, CE, OS, MD, KS, AB and QV had access to the raw data, verified and curated the data. LH and CE performed the formal analyses and created the visualizations. ML, TN and MW provided supervision. LH and CE drafted the original manuscript. ML, KS, MD, OS, AM, AB, QV, DG, MJ, TN and MW reviewed and edited the manuscript. LH and MW were responsible for the decision to submit the manuscript and subsequent revisions.

## Data sharing statement

Due to the different data-sharing policies of the various datasets included in this study, data included in this study will not be made publicly available. Requests for the data from each included dataset should be made to the corresponding author, The Netherlands Cancer Registry (NCR), the nationwide network and registry of histopathology and cytopathology (PALGA), and the Netherlands Organ Transplantation Registry (NOTR).

## Declaration of interests

MW served on Sanofi Genzyme's/Regeneron advanced cutaneous squamous cell carcinoma advisory board and was compensated. MW and CE presented this study at a Sanofi Genzyme internal meeting and were compensated. MJ was an advisory board member for Regeneron and Pierre Fabre. Payments for this work were made to the institute. TN is head of the dermatology department of the Erasmus MC. MW is chair of the European Taskforce Epidemiology of the EADV. AM was a member of the CSCC guideline committee and was paid for the contribution to the pathology section. AM is an advisory board member for HuKaS (patient organization for skin cancer) and the Stichting Melanoom (patient organization for melanoma), both unpaid. The remaining authors state no conflicts of interest.

## Appendix A Supplementary data

Supplementary Material

Dutch Summary
