# Supplementary material for: Longitudinal data on advanced cutaneous squamous cell carcinoma from the Dutch Keratinocyte Cancer Collaborative (DKCC): a nationwide real-world database study
Source: Lancet Reg Health Eur. 2025 Oct 18;59:101501. doi: 10.1016/j.lanepe.2025.101501 (PMC12556279; doi:10.1016/j.lanepe.2025.101501)
Supplement: Supplementary Material [file mmc1.docx]

**Supplementary Material**

Inhoud

[Supplementary methods 2](#_Toc201143902)

[Supplementary Table 1 Inclusion and exclusion criteria DKCC registry 5](#_Toc201143903)

[Supplementary Table 2. DKCC registration manual with core items for registration 6](#_Toc201143904)

[Supplementary figure 1 Value labels of item ‘topography extra’ 10](#_Toc201143905)

[Supplementary Table 3. Calculation of the estimated nationwide number of advanced CSCC 11](#_Toc201143906)

[Supplementary Table 4 Criteria for AJCC and BWH T-stage 12](#_Toc201143907)

[Supplementary Table 5 Estimated nationwide number of patients with advanced CSCC in the Netherlands in 2021. 16](#_Toc201143908)

[Supplementary Table 6 Data from figure 2: The Patient Journey – preceding CSCC stages 17](#_Toc201143909)

[Supplementary Table 7 Metastatic patterns until the worst type of metastasis 18](#_Toc201143910)

[Supplementary Table 8: Treatment details of primary and recurrent CSCC 19](#_Toc201143911)

[Supplementary Table 9 - Systemic treatment CSCC 20](#_Toc201143912)

# Supplementary methods

**Training of NCR registrars**

The training consisted of two parts. In the first part consisted of: general information about CSCC epidemiology; diagnosis and treatment of CSCC in clinical practice and the results of the pilot registration. In the second part, the registrars received detailed instructions how to register all items. Special attention was given to difficult to register items and situations for example: how to register multiple CSCC from the same patient, how to register items from the pathology report, how to register growth in the parotic gland, coding of metastasis, coding of radiotherapy items and Frequently Asked Questions (FAQ) that followed from the pilot registration. The training was recorded and was put on the NCR Sharepoint to enable registrars to lookup information during registration of new patients.

**Quality control of the registered data**

1 year after the start of the registration, a crosscheck of the data was performed. Two NCR registrars scored the following 9 items from the same patients (n=22). The following items were scored: date of incidence, morphology code, pathological T-stage, topography code, location of the lymph nodes, bone invasion, pathological tumoursize, tumour depth, largest diameter of the positive lymph node. For 1 item there was agreement for all registered patients, for 7 items there was agreement in 21/22 registered patients, for 1 item there was agreement in 20/22 registered items and for 2 items there was an interpretation difference for 2 or 3 patients. Lessons learned from this crosscheck were included in the DKCC manual to prevent similar errors in new registrations.

After the registration has been finalized quality controls were performed as well. This was performed when the datamanager or researcher observed odd situations. Examples include:

- when there was a large difference between the clinical and pathological diameter;

- when the tumour was registered as a T3/T4 tumour, but had no high risk histopathological features

- when the tumour was registered as a T1/T2 tumour but had high risk histopathological features

- when therapy response was missing, but the reason for stopping was a good or no response.

These quality checks remain possible in future analyses. When a researcher spots a discrepancy in the database, it is always possible for a NCR registrar to open the hospital files of the patient again to check (and correct, if needed) the registered items.

**Data selection for nationwide estimation of number locally advanced primary CSCC**

First, we selected all pathology reports of CSCC in Palga (>30,000). On this dataset we applied the pathology reports algorithm.^1^ All reports which were identified as potential T3/T4 AJCC 8 or T2b/T3 BWH tumours were selected (n=2,350).

To estimate the nationwide number of locally advanced CSCC, we focussed our manual review (by CE) on synoptic pathology reports (1,510 reports, 56%), as narrative reporting (1,146 reports, 44%) often lacked key tumour characteristics necessary for accurate T-staging.

We initially expected that locally advanced CSCC would be more frequently diagnosed in university medical centers. Therefore, we investigated the distribution of synoptic reports and free-text pathology reports, considering that a higher proportion of synoptic reports in university medical centers may lead to too high estimations of the nationwide number of locally advanced CSCC. However, we observed that synoptic reports were more frequently used in general hospitals. Specifically, only 14% of synoptic reports originated from academic hospitals compared to 40% of free-text pathology reports.

**Statistical analyses of nationwide estimations of number of patients and tumours per type of advanced CSCC**

For the different types of advanced CSCC, where virtually 100% of cases were registered (except for one center), we estimated the nationwide number patients using the sensitivity of the pathology algorithm (Supplementary Table 3).

First, we needed to correct for potentially missed patients, as one center in the Netherlands does not participate in the NCR. Registrations in this center were done separately and were not finished at the time of this study. Those patients will be included in future studies, but for the estimations of the nationwide number of patients we needed to take into account, that these patients were not included yet. We registered the number and type of advanced CSCCs treated in the non-participating center. We incorporated these tumours into the calculation of the estimated national number of patients after multiplying this number by the positive predictive value (PPV) of the algorithm (i.e., the percentage of all identified pathology reports that truly represent advanced cSCCs). The number of missed patients in the non-participating center was calculated as follows:

$$\frac{Number of identified patients by the pathology reports algorithm in 1 center}{Positive Predictive Value of the pathology reports algorithm} \times100\%$$

Thereafter, we added these numbers to the number of patients registered in DKCC. To calculate the nationwide number of patients with metastasis and recurrence, we applied the sensitivity of the pathology algorithm to the number of estimated tumours. The nationwide number of patients with metastasis and recurrence was then calculated as follows:

$$=\frac{number of registered patients + estimated number in 1 center}{sensitivity of the pathology reports algorithm}\times100\%$$

To calculate the nationwide occurrence of locally advanced CSCC, we also applied the sensitivity of the pathology reports algorithm. The number of advanced CSCC among synoptic pathology reports was calculated as follows:

$$= \frac{number of identified locally advanced tumours}{sensitivity of the pathology reports algorithm}\times100\%$$

Thereafter, we needed to extrapolate the number of primary locally advanced CSCC identified in synoptic reporting to the total number of pathology reports:

$$=\frac{incidence among synoptic reporting pathology reports}{Percentage of synoptic pathology reports} \times100\%$$

After we calculated the number of tumours, we also calculated among how many patients these tumours occurred. For this calculation we used the number of unique patients that were identified from the synoptic pathology reports that were manually read:

$$Number of patients=Number of tumours*correction factor$$

$$correction factor=\frac{Number of patients among 1,510 synoptic reports}{Number of tumours among 1,510 synoptic reports} \times100\%$$

95% Confidence intervals (CI) were not calculated according to the standard methodology for confidence intervals of proportions, because the total cohort size of recurrent and metastatic CSCC was unknown due to inclusion of both incident and prevalent cases. Instead, we used the 95% confidence intervals (CI) of the pathology algorithm’s sensitivity and PPV, reported in our previous publication to determine the 95% CI of the estimated nationwide number of patients. ^1^ All calculations for the estimated nationwide number of patients and tumours are detailed in Supplementary Table 3. For the calculation of 95% CI we repeated these calculation, but replaced the algorithms PPV and sensitivity with the lower and upper boundaries of those 95% CI. These values are reported in column E and H of Supplementary Table 3. For the calculation of the nationwide occurence of locally advanced CSCC, the PPV was not used, because all pathology reports that were identified on a nationwide level were manually read by CE, and the PPV (which represented the proportion of patients, that met the inclusion criteria of DKCC as determined by the NCR registrars who read the hospital files) was not relevant.

**Sensitivity analyses**

In the Netherlands, the AJCC 8 criteria are used, and the synoptic format consistently includes tumour invasion depth. However, it does not always specify whether the tumour extends beyond subcutaneous fat, which is a key criterion for BWH staging. To address this limitation, we conducted an additional analysis to identify tumours that met at least the T2a BWH classification but lacked only one risk factor – extension beyond the subcutaneous fat – to qualify as T2b. If the recorded invasion depth exceeded 6 mm, these tumours were also classified as T2b and considered primary locally advanced.

**References**

1. Eggermont C, Wakkee M, Bruggink A, Voorham Q, Schreuder K, Louwman M et al. Development and Validation of an Algorithm to Identify Patients with Advanced Cutaneous Squamous Cell Carcinoma from Pathology Reports. J Invest Dermatol 2022.

# Supplementary Table 1 Inclusion and exclusion criteria DKCC registry

| Description topography | Morphology | Topography ICD-O3 | A. Locally advanced primary (AJCC8) | B. Locally advanced primary (BWH) | | C. Local recurrence | D. N+ or M+ (including in-transit metastasis) |
| --- | --- | --- | --- | --- | --- | --- | --- |
| Included |  |  |  |  | |  |  |
| Skin head and neck, extremities, trunk and scrotum | 8010/3  8051/3  8070/3-8076/3 8078/3  8081/3  8083/3  8084/3 | C44.0 C44.2-4  C44.5-7 C63.2 | T3 or T4 | T2b/T3 | | Inclusion | Inclusion |
| Skin eyelid | Idem | C44.1 | T3 or T4 | T2b/T3 | | Inclusion | Inclusion |
| Vermillion border of upper- and lower lip, labial commissure | Idem | C00.0-1 C00.6 | T2, T3 or T4 | T2b/T3 | | Inclusion | Inclusion |
| Unknown primary location | Idem | C80.9 | Inclusion | Inclusion | | Inclusion | Inclusion |
| Excluded |  |  |  |  |  | |  |
| Skin vulva, penis and perianal region | Idem | C51, C60, C44.5 | Exclusion | Exclusion | Exclusion | | Exclusion |

Abbreviations: ICD-O3, third edition of the International Classification of Diseases for Oncology; AJCC8, American Joint Committee of Cancer eighth edition; BWH, Brigham and Women.

# Supplementary Table 2. DKCC registration manual with core items for registration

| **Patient characteristics** | |
| --- | --- |
| Age | at diagnosis for each tumour episode (primary tumour, recurrence, metastases) |
| Sex | Male/female |
| ~~Usage of immunosuppressive medication in the past~~ (see reason of exclusion below this table) | ~~No/yes/unknown~~ |
| ~~Usage of immunosuppressive medication during treatment~~ (see reason of exclusion below this table) | ~~No/yes/unknown~~ |
| Hematologic malignancy | Diagnosis date and type |
| WHO Performance (ECOG) status | WHO 0-1 good overall condition  WHO 2-3 not fit for palliative chemotherapy  WHO 3-4 spends almost entire day in bed |
| **Tumour characteristics** | |
| Clinical size | mm |
| Morphology | According to inclusion criteria, see Supplementary Table1 |
| Topography | According to inclusion criteria, Supplementary Table1 |
| Topography extra | Topography with detailed facial and neck areas (see supplementary figure 1) |
| Clinical TNM stage | cT/cN/cM |
| **Pathology characteristics – primary tumour or local recurrence** | |
| Pathological TNM stage | pT/pN/pM |
| Pathological size | In mm (or in case of synoptic reporting in pathology report: < 20 mm, 20-40 mm, ≥ 40 mm) |
| Differentiation | Good/moderate/poor or undifferentiated/unknown |
| Vertical tumour depth (measured from adjacent normal basement membrane to deepest point of tumour invasion) | In mm (or in case of synoptic reporting: > 6 mm) |
| Perineural invasion | No/yes/unknown |
| Perineural invasion extra | No perineural invasion/perineural invasion <0.1 mm/perineural invasion ≥ 0.1 mm /perineural invasion of a nerve deeper than the dermis/perineural invasion of unknown depth/unknown |
| Lymphovascular invasion | No/yes/unknown |
| Invasion beyond subcutaneous fat | No/yes/unknown |
| Invasion in muscle tissue | No/yes/unknown |
| Invasion in bone | No/minimal bone erosion/minimal cartilage invasion/invasion in base of skull/invasion in other bones or bone marrow/cartilage invasion/bone invasion or erosion of unknown category / unknown |
| ~~Mitosis~~ |  |
| **Regional lymph node metastases** | |
| Localization | Neck/axilla/superficial groin/deep groin/parotid/other |
| Diameter greatest lymph node clinically measured | In mm |
| Localization in relation to tumour | Ipsilateral/contralateral/bilateral/unknown |
| **Pathology characteristics – regional lymph node metastasis** | |
| Number of nodes removed |  |
| Number of positive nodes removed |  |
| Diameter greatest lymph node pathologically measured | In mm |
| Extranodal extension | Yes/no/unknown |
| **In-transit metastases** | |
| Metastasectomy skin |  |
| Radiotherapy in-transit metastasis |  |
| In-transit metastasis localizations |  |
| **Distant metastases** | |
| Number of distant metastasis |  |
| Distant metastases localizations |  |
| **Radiology and imaging** | |
| Reason for imaging | Symptoms/surveillance/incidental finding/unknown |
| Type of imaging | Ultrasound/CT/CT brain/CT thorax/MRI/MRI brain/PET-CT/PET-MRI |
| **Treatments** | |
| All treatments | Start date and end date |
| Reason no treatment | Comorbidity/functional status/age/died shortly after diagnosis/patient wish or refusal/high tumour burden, too extensive disease or progression/other/unknown |
| ***Surgical treatments*** |  |
| **Surgical treatments – primary tumour or recurrence** | Excision, mohs micrographic surgery, breuninger (slow-mohs), amputation, orbital exenteration, additional bone resection/frasing of the bone, curettage and shave, photodynamic therapy, cryosurgery. |
| Resection margin status | Microscopic complete resection/microscopic incomplete resection/macroscopic incomplete resection |
| ***Radiation*** |  |
| Type of radiotherapy | Radiation on primary tumour/radiation on primary tumour and involved lymph nodes/radiation to involved lymph nodes/ elective radiation to lymph nodes/internal radiotherapy (brachytherapy) |
| Intention | Curative/Palliative/Unknown |
| Dosage | Number of factions, dose per fraction, and total dose (Gy) |
| ***Systemic therapy*** |  |
| Systemic therapy | Chemotherapy/eGFR-inhibitors/Immunotherapy |
| Reason stop systemic treatment | Not stopped/comorbidity/functional status/patients wish or refusal/progression or no response/good therapeutic response/unknown or other |
| Treatment response | Complete response/progressive disease/partial response/stabile disease |
| Complications following systemic treatment | No serious complication/> grade 3 |
| **Follow-up**^a^ | |
| End of clinical follow-up | Date |
| Tumour status^b^ | Disease-free/(residual) tumour with initiation of tumour-directed treatment/(residual)tumour without tumour-directed treatment/unknown |
| Vitale status | Alive/deceased |

^a^ Administrative follow-up is conducted annually by the dedicated DKCC team of NCR registrars.

^b^ In cases of tumour progression (local recurrence or metastases), a new tumour episode is created by the registrars, and all items for this subsequent episode are re-registered.

**Removed items after feasibility study**

- Use of immunosuppressive medication in the past (no/yes/unknown)
- Use of immunosuppressive medication during treatment (no/yes/unknown)

We removed these items from the registration manual because their classification as “immunosuppressive” lacked precision, as it depends on the type, dose and duration of treatment. Capturing this level of detail would require excessive registration time from the registrars. To better/more efficiently analyze the impact of immunosuppressive therapy on CSCC disease progression and mortality, we aim to link our data with Vektis – a comprehensive Dutch nationwide database of healthcare claim. This will provide complete medication histories, including dosage and duration, while reducing workload for registrars.

- Mitosis

This item was removed due to a very high proportion of missing values (85%) during the pilot registration phase.

- Distinction between intraparotid metastases and metastasis in parotid gland lymph nodes

Differentiating between metastases within the parotid gland and those in its lymph nodes was found to be too complex for data managers, as it relied on pathology reports that did not always provide clear differentiation.

# Supplementary figure 1 Value labels of item ‘topography extra’

**
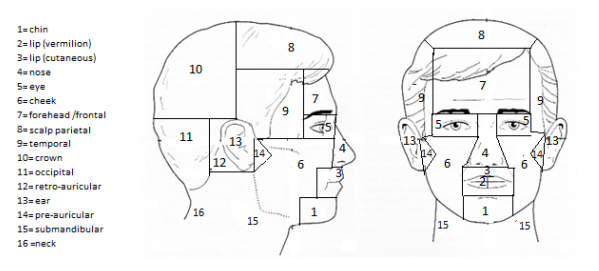
**Anatomic location with detailed face and neck areas.

# Supplementary Table 3. Calculation of the estimated nationwide number of advanced CSCC

| A | B | C | D | E | F | G | H | I |
| --- | --- | --- | --- | --- | --- | --- | --- | --- |
| Type of advanced cSCC | **Number of patients registered in DKCC** | **Identified tumours from pathology reports with synoptic reporting (n)** | **% Synoptic reporting** | **Sensitivity pathology reports algorithm (95% CI)** | **Correction factor^b^** | **Missed registrations non-participating center^c^** | **Positive predictive value pathology reports algorithm** | **Calculation nationwide incidence** |
| AJCC8 T3/T4 | N/A | 889 | 56% | 86.2% (79.9-92.5) | 0.95 | N/A | N/A | C/D/E*F |
| BWH T2b/T3 | N/A | 291^a^ | 56% | 52.3% (40.1-64.5) | 0.95 | N/A | N/A | C/D/E*F |
| recurrent CSCC | 118 | N/A | N/A | 23.3% (8.2-38.5) | N/A | 3 | 78.6% (63.3-93.8) | (B + (G*H))/E |
| metastatic CSCC | 171 | N/A | N/A | 70.0% (55.8-84.2) | N/A | 14 | 62.4% (54.2-70.7) | (B + (G*H))/E |

Abbreviations: CSCC, cutaneous squamous cell carcinoma; PPV, positive predictive value; CI, confidence interval; AJCC8, American Joint Committee on Cancer, eighth edition; BWH, Brigham and Women.

^a^If we assume invasion depth > 6 mm as to represent invasion beyond the subcutaneous fat, the count of tumours classified as T2b/T3 according to BWH criteria would increase to 593.

^b^ tumourA correction factor was applied to determine the number of patients with locally advanced primary tumours. The calculation of the correction factor is described in the supplementary methods.

^c^In the Netherlands, one medical center that treats CSCC patients is not linked to the Netherlands Cancer Registry, so patients treated there were not reviewed by NCR registrars. To adjust, we estimated the nationwide incidence by multiplying the patient count from this center by the algorithm’s positive predictive value.

# Supplementary Table 4 Criteria for AJCC and BWH T-stage

| **T stage** | **Criteria** | **Variables included in DKCC to assign T-stage (From Supl Tab. 2)** | **Values to assign the T-stage** | **Values not taken into account as high risk features for this T-stage** |
| --- | --- | --- | --- | --- |
| **AJCC** | | | | |
| T1 | Tumour diameter≤ 2 cm | Clinical size in mm  If clinical size was missing Pathological size was used | ≤ 20 mm |  |
| T2 | Tumour diameter>2 cm and ≤ 4 cm | Clinical size in mm  If clinical size was missing Pathological size was used | >20 mm and ≤ 40 mm |  |
| T3 | Tumour diameter>4 cm | Clinical size in mm  If clinical size was missing Pathological size was used | >40 mm |  |
|  | Minimal bone erosion | Invasion in bone | Minimal bone erosion | -Minimal cartilage invasion  -Cartilage invasion  -Bone invasion or erosion of unknown category  -Unknown |
|  | Perineural invasion (in nerves > 0.1 mm or in deep nerves (>dermis) | Perineural invasion extra | -Perineural invasion ≥ 0.1 mm  -Perineural invasion of a nerve deeper than the dermis  -Perineural invasion of unknown depth | -No perineural invasion  -Perineural invasion <0.1 mm |
|  | Deep invasion (>6 mm or beyond the subcutaneous fat) | Vertical tumour depth (measured from adjacent normal basement membrane to deepest point of tumour invasion) | >6 mm |  |
|  |  | Invasion beyond subcutaneous fat | Yes | No/unknown |
|  |  | Invasion in muscle tissue | Yes  (in case of bone/cartilage invasion or erosion and unknown invasion in muscle, muscle invasion was considered ‘yes’) | No/unknown |
| T4a | Invasion in bone/bone marrow | Invasion in bone | Invasion in other bones or bone marrow | -Minimal cartilage invasion  -Cartilage invasion  -Bone invasion or erosion of unknown category  -Unknown |
| T4b | Tumour invasion in the skull | Invasion in bone | Invasion in base of skull | -Bone invasion or erosion of unknown category  -Unknown |
| **BWH** | | | | |
| High-risk factors: | Tumour diameter ≥ 2 mm | Clinical size in mm  If clinical size was missing Pathological size was used | ≥ 20 mm |  |
|  | Poor differentiation | Differentiation | Poor or undifferentiated | Good/Moderate/Unknown |
|  | Perineural invasion ≥ 0.1 mm | Perineural invasion extra | -Perineural invasion ≥ 0.1 mm  -Perineural invasion of a nerve deeper than the dermis  -Perineural invasion of unknown depth | -No perineural invasion  -Perineural invasion <0.1 mm |
|  | Tumour invasion beyond the subcutaneous fat | Invasion beyond subcutaneous fat | Yes | No/unknown |
| T1 | 0 Risk factors | Sum of aforementioned high-risk factors |  |  |
| T2a | 1 High-risk factor | Sum of aforementioned high-risk factors |  |  |
| T2b | 2-3 High-risk factors | Sum of aforementioned high-risk factors |  |  |
| T3 | ≥ 4 High-risk factors | Sum of aforementioned high-risk factors |  |  |
|  | Bone invasion |  | -Invasion in other bones or bone marrow  -Invasion in base of skull | -Minimal bone erosion  -Minimal cartilage invasion  -Cartilage invasion  -Bone invasion or erosion of unknown category  -Unknown |

# Supplementary Table 5 Estimated nationwide number of patients with advanced CSCC in the Netherlands in 2021.

|  | Included in the DKCC  (N, 2021) | Nationwide incidence of primary CSCC | % |  |
| --- | --- | --- | --- | --- |
| Incidence CSCC (new patients) |  | 14,700 | N/A |  |
| All first and subsequent CSCCs |  | 23,065 | 100 |  |
| Total number of patients in DKCC | 503 | N/A |  |  |
|  |  |  |  |  |
| *Registration of all patients in the Netherlands in DKCC* | | | |  |
|  | **Identified patients from the DKCC** | **Nationwide estimation of number of patients (95% CI)^a^** | **% (95% CI)^a^** |  |
| Recurrent CSCC | 118 | 521 (311-1500) | N/A |  |
| Metastatic CSCC | 171 | 257 (214-321) | N/A |  |
| *Registration of a random sample of all patients in the Netherlands in DKCC* | | | |  |
|  | **Identified tumours and patients from all synoptic pathology reports in the Netherlands (N, 2021)** | **Nationwide estimation of number of tumours and patients (95% CI)^a^** | **% (95% CI)^a^** |  |
| Primary locally advanced AJCC 8 T3/T4 | | | |  |
| *Patients* | 828 | 1754 (1621-1885) | N/A |  |
| *Tumours* | 889 | 1846 (1707-1984) | 8.0 (7.4-8.6) |  |
| Primary locally advanced BWH T2b/T3 | | | |  |
| *Patients* | 258 | 949 (759-1234) | N/A |  |
| *Tumours* | 291 | 999 (799-1299) | 4.3 (3.5-5.6) |  |
| *Sensitivity Analyses* |  |  |  |  |
| Primary locally advanced BWH T2b/T3, assuming >6 mm invasion depth is beyond subcutaneous fat | | | |  |
| *Patients* | 541 | 1934 (1547-2514) | N/A |  |
| *Tumours* | | 593 | 2036 (1629-2647) | 8.8 (7.1-11.5) |
|  |  |  |  |  |

Abbreviations: CSCC, cutaneous squamous cell carcinoma; CI, confidence interval; AJCC 8, American Joint Committee on Cancer, eighth edition; BWH, Brigham and Women; N/A, not applicable.

^a^ The 95% CI in this table were not calculated based on standard methodology, but were based on the 95% CI of the sensitivity of the algorithm to identify advanced CSCC from the pathology reports (Eggermont et al, JID, 2023)

# Supplementary Table 6 Data from figure 2: The Patient Journey – preceding CSCC stages

| Which type of CSCC occurred before the second CSCC episode? | | | |
| --- | --- | --- | --- |
| Second CSCC episode | **First CSCC episode** | **N** | **%** |
| Alive, no second episode (n=342) | T3/T4 | 297 | 87% |
|  | Metastasis | 45 | 13% |
|  |  |  |  |
| Recurrence (n=164) | T1/T2 | 91 | 55% |
|  | T3/T4 | 69 | 42% |
|  | Metastasis | 4 | 2% |
|  |  |  |  |
| Metastasis (n=265) | T1/T2 | 121 | 46% |
|  | T3/T4 | 123 | 46% |
|  | Metastasis | 21 | 8% |
|  |  |  |  |
| Death, any cause (n=78) | T1/T2 | 6 | 8% |
|  | T3/T4 | 54 | 69% |
|  | Metastasis | 18 | 23% |
|  |  |  |  |
| Which type of CSCC occurred before the third CSCC episode? | | | |
| Third episode | **Second episode** | **N** | **%** |
| Alive, no third episode (n=235) | Recurrence | 85 | 36% |
|  | Metastasis | 150 | 64% |
|  |  |  |  |
| Recurrence (n=58) | Recurrence | 49 | 84% |
|  | Metastasis | 9 | 16% |
|  |  |  |  |
| Death, any cause (n=84) | Recurrence | 25 | 30% |
|  | Metastasis | 59 | 70% |
|  |  |  |  |
| Metastasis (n=76) | Recurrence | 19 | 25% |
|  | Metastasis | 57 | 75% |

# Supplementary Table 7 Metastatic patterns until the worst type of metastasis

|  | Sequence of events |  |  |  |
| --- | --- | --- | --- | --- |
| 361 | **Total number of patients** |  |  |  |
| 84 (23%) | **Patients with metastasis at diagnosis** |  |  |  |
| N | **First diagnosis** | **Second episode** | **Third episode** | **Fourth episode** |
| 5 | Skin metastasis |  |  |  |
| 64 | Lymph node metastasis |  |  |  |
| 11 | Distant metastasis |  |  |  |
| 1 | Skin metastasis | Lymph node metastasis | Distant metastasis |  |
| 3 | Lymph node metastasis | Distant metastasis |  |  |
|  | | | | |
| 277 (77%) | **Patients with metastasis during FU** |  |  |  |
| N | **First diagnosis** | **Second episode** | **Third episode** | **Fourth episode** |
| 24 | Primary tumour without metastasis | Skin metastasis |  |  |
| 174 | Primary tumour without metastasis | Lymph node metastasis |  |  |
| 28 | Primary tumour without metastasis | Distant metastasis |  |  |
| 3 | Primary tumour without metastasis | Skin metastasis | Lymph node metastasis |  |
| 1 | Primary tumour without metastasis | Skin metastasis | Distant metastasis |  |
| 19 | Primary tumour without metastasis | Lymph node metastasis | Distant metastasis |  |
| 2 | Primary tumour without metastasis | Lymph node metastasis | Skin metastasis | Distant metastasis |
| 19 | Primary tumour without metastasis | Recurrent CSCC | Skin or lymph node metastasis |  |
| 2 | Primary tumour without metastasis | Recurrent CSCC | Skin or lymph node metastasis | Distant metastasis |
| 5 | Primary tumour without metastasis | Recurrent CSCC | Distant metastasis |  |

Remark: Patients can have a recurrent CSCC during the same episode with metastasis. Those recurrences are not reported in this table. Only the metastasis is reported in that case. This table only reports on episodes with only a recurrent CSCC. In Table 2 it is reported how many patients have a recurrence at the same time as metastases.

# Supplementary Table 8: Treatment details of primary and recurrent CSCC

|  | Primary locally advanced CSCC | | Recurrent SCC | |
| --- | --- | --- | --- | --- |
|  | AJCC8 T3/T4 | BWH T2b/T3 | Primary CSCC of the recurrence | Recurrence |
|  | **n = 559** | **n = 278** | **n=198^a^** | **n = 263** |
| Treated with excision | 507 | 248 | 184 | 197 |
| Number of excisions: |  |  |  |  |
| 1 | 302 (60%) | 145 (58%) | 104 (57%) | 142 (72%) |
| 2 | 158 (31%) | 73 (29%) | 40 (22%) | 39 (20%) |
| ≥3 | 28 (6%) | 18 (7%) | 12 (7%) | 10 (5%) |
| Unknown number of excisions | 19 (4%) | 12 (5%) | 29 (16%) | 6 (3%) |
|  |  |  |  |  |
| Positive margins after last excision | 131 | 71 | 51 | 56 |
| Additional Treatment: | 42 | 18 | 10 | 23 |
| Adjuvant RT | 29 (69%) | 14 (78%) | 7 (70%) | 13 (57%) |
| Systemic treatment | 0 (0%) | 0 (0%) | 1 (10%) | 4 (17%) |
| Margin-controlled surgery | 10 (24%) | 2 (11%) | 2 (20%) | 2 (9%) |
| Major surgery | 1 (2%) | 1 (6%) | 0 (0%) | 3 (13%) |
| Margin-controlled surgery or major surgery + RT | 2 (5%) | 1 (6%) | 0 (0%) | 1 (4%) |
|  |  |  |  |  |

Abbreviations: CSCC, cutaneous squamous cell carcinoma; AJCC 8, American Joint Committee of Cancer, 8th edition; BWH, Brigham and Women Hospital RT, radiotherapy; NA, not applicable.

^a^ As tumours may recur multiple times, the number of primary tumours (n=198) is lower than the number of recurrence episodes (n=263).

# Supplementary Table 9 - Systemic treatment CSCC

|  | Cemiplimab  N | Other systemic treatment  N |
| --- | --- | --- |
| Type of episode |  |  |
| Primary CSCC, T3/T4 | 0 | 3 |
| Recurrent CSCC | 7 | 3 |
| Skin metastasis | 5 | 0 |
| Regional Lymphnode metastasis | 12 | 8 |
| Distant metastasis | 24 | 4 |
|  |  |  |
| Type of other systemic treatment |  |  |
| Local chemotherapy, NOS |  | 2 |
| Gemcitabine/cisplatine |  | 1 |
| Cetuximab |  | 1 |
| Nivolumab |  | 1 |
| Pembrolizumab |  | 1 |
| Atezolizumab |  | 1 |
| Cisplatine |  | 1 |
| Carboplatine |  | 1 |
| Ipilimumab/nivolumab |  | 4 |
| Paclitaxel/carboplatine/pembrolizumab |  | 1 |
| Melfalan/TNF-alpha inhibitors |  | 1 |
| Type of other systemic treatment |  | 2 |
| Local chemotherapy, NOS |  | 1 |
| Gemcitabine/cisplatine |  | 1 |
| Cetuximab |  | 1 |
| Nivolumab |  | 1 |
| Pembrolizumab |  | 1 |
|  |  |  |
| Treatment response |  |  |
| CR (complete response) | 2 | 3 |
| PR (partial response) | 12 | 2 |
| SD (stabile disease) | 2 | 0 |
| PD (progressive disease) | 2 | 2 |
| Not determined | 3 | 0 |
|  |  |  |
| Adverse effect | 4 | 0 |
|  |  |  |
| Stopped Treatment | 15 | 1 |
| Reason for stopping treatment |  |  |
| Comorbidity | 0 | 0 |
| Functional status | 1 | 0 |
| Refused treatment patient or family | 3 | 0 |
| Progression / No response | 7 | 1 |
| Good response to therapy | 4 | 0 |
|  |  |  |

Abbreviations: CSCC, cutaneous squamous cell carcinoma; NOS Not otherwise Specified
