# Supplementary material for: Longitudinal data on advanced cutaneous squamous cell carcinoma from the Dutch Keratinocyte Cancer Collaborative (DKCC): a nationwide real-world database study
Source: Lancet Reg Health Eur. 2025 Oct 18;59:101501. doi: 10.1016/j.lanepe.2025.101501 (PMC12556279; doi:10.1016/j.lanepe.2025.101501)
Supplement: Dutch Summary [file mmc2.docx]

# Editor disclaimer: This translation in Dutch was submitted by the authors and we reproduce it as supplied. It has not been peer reviewed. Our editorial processes have only been applied to the original abstract in English, which should serve as reference for this manuscript.

Achtergrond: Het plaveiselcelcarcinoom (PCC) van de huid is één van de meest voorkomende vormen van kanker, maar epidemiologische gegevens zijn schaars. Het doel van deze studie was daarom om hoogwaardige epidemiologische gegevens te verzamelen, die nodig zijn om verschillen in uitkomsten in kaart te brengen en de patiëntenzorg te verbeteren. Om dit doel te bereiken, hebben we de Dutch Keratinocyte Cancer Collaborative (DKCC) opgezet, een landelijke database met longitudinale gegevens over lokaal vergevorderd, recidiverend en gemetastaseerd PCC (vergevorderd PCC).

Methoden: Vergevorderde PCC's werden geïdentificeerd uit de Nederlandse landelijke Pathologie Databank (Palga) met behulp van een gevalideerd algoritme. Medewerkers van de Nederlandse Kankerregistratie registreerde een selectie van 500 patiënten per kalenderjaar met vergevorderd PCC (alle gemetastaseerde, alle recidieven en een willekeurige selectie van lokaal vergevorderde PCC's). Gegevens over tumorkenmerken, diagnostiek, ziekteprogressie en behandeling werden geregistreerd. De gegevens werden gekoppeld aan het Nederlands Orgaantransplantatie Register om informatie te verkrijgen over immuungecompromiteerde patiënten en gekoppeld aan de gemeentelijke basisadministratie voor de vitale status.

Resultaten: Op basis van de geregistreerde gegevens, schatten we dat 8,0% (1.846/23.065 PCC’s, 95%-betrouwbaarheidsinterval: 7,4-8,6) van alle gediagnosticeerde PCC’s in 2021 een lokaal vergevorderd PCC was. In 2021-2022 werden 920 patiënten met vergevorderd PCC geregistreerd in de DKCC database. Een kwart van de gemetastaseerde patiënten had een recidief PCC voordat er metastasen ontstonden (d.w.z. 13/51 patiënten met huidmetastasen, 63/296 patiënten met regionale lymfekliermetastasen, 20/72 patiënten met metastasen op afstand). De mediane tijd tot recidief of metastase na een T3/T4 primair PCC van de American Joint Committee on Cancer (AJCC) was respectievelijk 11 maanden (interkwartielafstand [IQR] 6-19) en 9 maanden (IQR 6-17). Bij 6% (4/67, alleen huidmetastasen) tot 20% (17/83, metastasen op afstand) van de episodes met metastasen werd geen behandeling gegeven.

Interpretatie: De DKCC database is de eerste landelijke longitudinale databron over vergevorderd PCC. De methodologie kan dienen als voorbeeld voor het ontwerpen van efficiënte registraties voor vergevorderd PCC in andere landen of zelfs voor andere zeldzame kankeruitkomsten. Het hoge aantal lokaal vergevorderde PCC’s zorgt voor een grote druk op de gezondheidszorg, omdat deze patiënten uitgebreidere diagnostiek en behandeling nodig hebben. Gegevens van de DKCC databank bieden essentiële informatie om klinische richtlijnen voor optimale PCC-patiëntenzorg te verbeteren.

Financiering: Dit onderzoek werd gefinancierd door Sanofi Genzyme/Regeneron.
